# The Prognostic Role of C-Reactive Protein Velocity in Patients with First Acute Myocardial Infarction

**DOI:** 10.3390/jcm14217633

**Published:** 2025-10-28

**Authors:** Stylianos Daios, Vasileios Anastasiou, Dimitrios V. Moysidis, Matthaios Didagelos, Andreas S. Papazoglou, Christos Gogos, Nikolaos Stalikas, Efstratios Alexiadis, Konstantinos C. Theodoropoulos, Eleftheria Ztriva, Georgia Kaiafa, Kali Makedou, Vasileios Kamperidis, Antonios Ziakas, Christos Savopoulos

**Affiliations:** 1 First Department of Cardiology, AHEPA University Hospital, School of Medicine, Faculty of Health Sciences, Aristotle University of Thessaloniki, 54124 Thessaloniki, Greece; stylianoschrys.daios@gmail.com (S.D.); vasianas44@gmail.com (V.A.); manthosdid@yahoo.gr (M.D.); cgogos.cardio@gmail.com (C.G.); ealexiadi@auth.gr (E.A.); ktheod2005@hotmail.com (K.C.T.); vkamperidis@outlook.com (V.K.); tonyziakas@hotmail.com (A.Z.); 2 Medical School, Aristotle University of Thessaloniki, 54124 Thessaloniki, Greece; dimoysidis@gmail.com; 3 Athens Naval Hospital, 11521 Athens, Greece; anpapazoglou@yahoo.com; 4 Cardiovascular Center Aalst, AZORG Ziekenhuis, 9300 Aalst, Belgium; nstalik@gmail.com; 5 First Propaedeutic Department of Internal Medicine, University General Hospital of Thessaloniki AHEPA, Aristotle University of Thessaloniki, 54636 Thessaloniki, Greece; elztriva@gmail.com (E.Z.); gdkaiafa@yahoo.gr (G.K.); 6 Laboratory of Biochemistry, AHEPA University Hospital, School of Medicine, Aristotle University of Thessaloniki, 54124 Thessaloniki, Greece; kmakedou@auth.gr

**Keywords:** C-reactive protein, CRPv, acute myocardial infarction, inflammation, biomarkers, prognosis

## Abstract

**Background/Objectives**: Inflammation plays a key role in the pathophysiology of acute myocardial infarction (AMI). Yet static measures of C-reactive protein (CRP) provide limited prognostic information. CRP velocity (CRPv), which reflects the rate of CRP rise within the first 24 h, may better depict the dynamic inflammatory response. To investigate the prognostic role of CRPv in patients presenting with a first AMI. **Methods**: Consecutive patients presenting with first AMI were enrolled. CRPv was calculated as the difference between CRP at admission and after 24 ± 8 h, divided by time. A prognostic CRPv cut-off was derived from spline curve analysis to dichotomize the population. Patients were followed up for the primary composite endpoint of cardiovascular death, non-fatal AMI, and hospitalization for heart failure. **Results**: Among 604 patients, 189 (31.3%) had CRPv ≥ 1.36 mg/L/h and 415 (68.7%) had CRPv < 1.36 mg/L/h. Higher hs-cTnT (adjusted odds ratio [aOR] 2.552, 95% CI, 1.520–4.286; *p* < 0.001) and NT-proBNP (aOR 2.229, 95% CI, 1.241–4.002; *p* = 0.007) were independently associated with CRPv ≥ 1.36 mg/L/h. At a median follow-up of 13.8 months, 115 patients (19.0%) reached the primary composite endpoint. High CRPv patients had significantly lower event-free survival rate than low CRPv patients (66.7% vs. 85.5%, log-rank *p* < 0.001). CRPv independently predicted the primary composite endpoint [adjusted hazard ratio 1.226, 95% CI 1.102–1.364, *p* < 0.001]. Adding CRPv on top of clinical, echocardiographic, and biochemical risk factors significantly improved model discrimination (*p* < 0.001), whereas single CRP on admission (*p* = 0.947) or CRP 24 ± 8 h from admission (*p* = 0.064) did not. **Conclusions**: CRPv appears to be a robust predictor of adverse outcomes in first AMI patients, offering incremental prognostic value beyond established clinical and biomarker indices. Its feasibility and low cost support its integration into early clinical risk stratification.

## 1. Introduction

Inflammation is pivotal to the pathophysiology of acute myocardial infarction (AMI), influencing both the extent of initial myocardial damage and subsequent complications [1]. C-reactive protein (CRP) is a well-validated marker of systemic inflammation that has been extensively studied in cardiovascular diseases and, in particular, AMI [1,2]. CRP levels begin to rise within hours of symptom onset, peak at 24–48 h post-AMI, and have been linked to multiple adverse outcomes, such as left-ventricular dysfunction, arrhythmias, acute kidney injury and microvascular obstruction [3]. Yet, a single CRP measurement on admission provides only a snapshot of an evolving inflammatory process and fails to reflect the dynamic inflammatory response that occurs after AMI.

Multiple studies have highlighted the value of CRP in predicting adverse outcomes including heart failure hospitalization and mortality [4]. While accumulating evidence supports the dynamic nature of CRP levels assessed at different time intervals, uncertainty remains regarding the optimal timing for CRP measurement following symptom onset. Therefore, assessing the early fluctuations and temporal trends of CRP may be a more sensitive and reliable approach to depict the extent of a patients’ inflammatory response and the risk of future cardiovascular events compared to a single static measurement at one time point [5]. This concept is effectively represented by CRP velocity (CRPv), a dynamic biomarker reflecting the rate of CRP rise that is calculated as the difference between the CRP concentration at hospital admission and the measurement obtained after 24 ± 8 h, divided by the elapsed time between these two measurements [6].

The aim of this study was to examine the prognostic role of CRPv in patients with first AMI and investigate its added value on top of established risk factors in AMI.

## 2. Methods

### 2.1. Study Design and Population Sources

This study is an analysis derived from the “CLEAR-AMI study” (ClinicalTrials.gov Identifier: NCT05791916), a prospective, non-interventional cohort study enrolling consecutive patients admitted to the Cardiology Department of AHEPA University General Hospital in Thessaloniki, Greece, from March 2022 to July 2024, with a diagnosis of first AMI, including both ST-elevation myocardial infarction and non-ST-elevation myocardial infarction [7]. The study protocol received approval from the Ethics Committee of the Aristotle University of Thessaloniki (reference number: 6.582/2022) and complies with the ethical standards described in the Declaration of Helsinki (2013 amendment). Informed written consent was obtained from each patient at enrollment.

All patients underwent percutaneous coronary intervention of the culprit lesion, according to the current guidelines [8]. Interventional and pharmacological treatment for AMI were applied consistently following the relevant guidelines [8]. On admission, the data that were collected included accurate timing of symptom onset, Global Registry of Acute Coronary Events (GRACE) risk score, as well as anthropometric, biochemical, echocardiographic and clinical parameters. Patients with a known medical history of heart failure, cardiomyopathy, severe valvular heart disease, or pulmonary hypertension were excluded from participation. Patients with diagnosed concomitant infection were excluded from the analysis.

In all patients, blood samples for CRP were drawn upon admission at the emergency department or in the catheterization laboratory (CRPadm), prior to percutaneous coronary intervention. A second CRP measurement (2ndCRP) was obtained following primary percutaneous coronary intervention, and within 24 ± 8 h from admission. CRPv was calculated as the difference between 2ndCRP (mg/L) and CRPadm (mg/L), divided by the time interval (in hours) that elapsed between the two measurements.

### 2.2. Follow-Up

Patients were followed up through biannual clinic visits or telephone contact with the patients or their immediate relatives. Clinical endpoints were defined according to the ESC guidelines criteria [9]. They were reviewed by independent investigators who were blinded to the recruitment procedure and to the CRPv values. The primary endpoint was a composite of cardiovascular death, non-fatal AMI and hospitalization for heart failure. If a patient experienced several events during the follow-up period, only the first was counted in the calculation of events’ occurrence. Patient enrollment began in March 2022. The median duration of follow-up was 13.8 (8.4–18.7) months. There were no missing follow-up data.

### 2.3. Statistical Analysis

Baseline characteristics of the study population were analyzed according to CRPv. Spline curve analysis was performed by assessing the hazard ratio (HR) for the primary composite endpoint across the range of CRPv values. The CRPv value for which the HR of the lower limit of the 95% confidence intervals (CI) was ≥1 was used as a prognostic cut-off value for sample dichotomization.

Comparisons between groups were performed using the chi-squared (χ^2^) test for categorical variables and the two-sided Student’s t-test for continuous variables. In cases where the assumption of normality was not met, the non-parametric Mann–Whitney U test was employed instead. Categorical data were reported as absolute frequencies and percentages, while continuous variables were expressed as mean ± standard deviation or median with interquartile range.

Relevant clinical, laboratory, and echocardiographic variables were tested using univariable logistic regression analysis to identify associations with high CRPv. Variables that demonstrated statistical significance in the univariable analysis were entered into a multivariable model to find independent associations. The adjusted odds ratios along with 95% CIs and *p*-values were calculated and reported.

A time-to-first-event analysis was performed to examine the relationship between CRPv and the primary composite endpoint. Participants were censored either at the occurrence of the event or at their last documented interaction with the study investigator. Event rates were illustrated using Kaplan–Meier survival curves and statistically compared using the log-rank test.

Univariable Cox proportional regression analyses were performed to identify significant predictors of the primary composite endpoint among the clinically relevant parameters. All univariably significant parameters were entered in a multivariable Cox proportional regression model, to identify independent predictors of the primary composite endpoint.

The incremental prognostic value of CRPv was evaluated using nested Cox models. A baseline clinical model was generated including all the variables that demonstrated significant association with the primary composite endpoint in univariable Cox regression analysis. Subsequently all the statistically significant echocardiographic and laboratory risk factors were added on top as next steps. As final step, CRPadm, 2ndCRP and CRPv were separately added on top of the existent model. Improvements in model fit were quantified by the change in chi-square (χ^2^) statistics.

A two-sided *p*-value of less than 0.05 was considered statistically significant for all tests. Results were reported with 95% CIs. Data processing and statistical analyses were performed using IBM SPSS Statistics version 26 and R version 3.4.4 (R Foundation for Statistical Computing, Vienna, Austria).

## 3. Results

**Figure 1:**
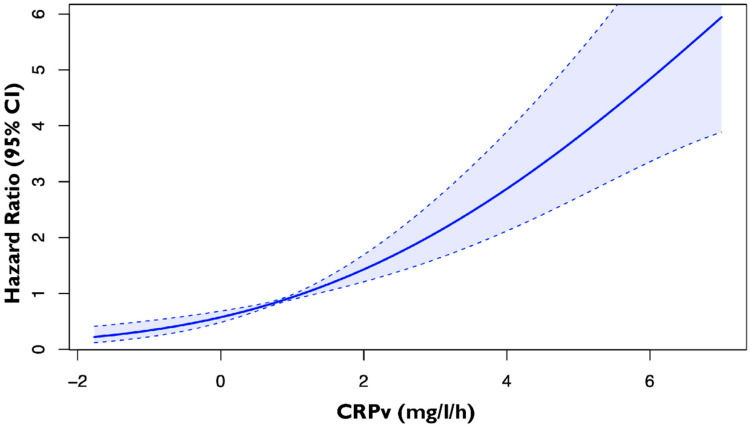
Spline curve by plotting the hazard ratio of the primary composite endpoint of cardiovascular death, non-fatal MI and hospitalization for heart failure according to CRPv in patients with first AMI. The bold blue line represents the spline curve, with overlaid 95% confidence intervals illustrated (light blue areas). Abbreviations: CRPv, C-reactive protein velocity; MI, myocardial infarction; CI, confidence interval.

A total of 611 consecutive patients admitted to the hospital with first AMI were prospectively enrolled. Assessment of CRPv was feasible in a total of 604 patients (mean age 62.0 ± 11.8 years, male 78.9%) who were included in this analysis. These were divided into 2 groups based on the CRPv prognostic cutoff value that was derived from the spline curve analysis (Figure 1); patients with CRPv ≥ 1.36 mg/L/h (n = 189, 31.3%) and CRPv < 1.36 mg/L/h (n = 415, 68.7%). The clinical, biochemical and echocardiographic characteristics for the 2 groups are presented in Table 1. Among others, patients with CRPv ≥ 1.36 mg/L/h were older, had higher GRACE risk scores and were more likely to have a history of type 2 diabetes mellitus. Regarding their echocardiographic and laboratory indices they had lower LVEF and TAPSE and higher hs-cTnT and NT-proBNP.

### 3.1. Associates of CRPv ≥ 1.36 mg/L/h

Table 2 summarizes all the relevant clinical, biochemical, and echocardiographic variables associated with CRPv ≥ 1.36 mg/L/h in univariable and multivariable logistic regression analysis. Higher values of hs-cTnT (adjusted odds ratio 2.552 [95% CI, 1.520–4.286; *p* < 0.001) and NT-proBNP (adjusted odds ratio 2.229 [95% CI, 1.241–4.002; *p* = 0.007) were independently associated with CRPv ≥ 1.36 mg/L/h.

### 3.2. Outcome Analysis

During a median follow-up of 13.8 (8.4–18.7) months, 115 patients (19.0%) reached the primary composite endpoint. Table 3 summarizes the clinical, biochemical and echocardiographic variables that demonstrated independent association with the primary endpoint. Several clinical, biochemical, and echocardiographic parameters were tested with univariable Cox regression analysis and only those that demonstrated significant association with the primary composite outcome were entered in the multivariable analysis. Regarding the clinical parameters, GRACE risk score (aHR 1.012 [95% CI, 1.003–1.021; *p* = 0.009) and type 2 diabetes mellitus (aHR 1.692 [95% CI, 1.005–2.847; *p* = 0.048) retained independent association with the primary composite endpoint. With regard to echocardiographic and biochemical parameters, LVEF (aHR 0.959 [95% CI, 0.933–0.987; *p* = 0.004) and CRPv (aHR 1.226 [95% CI, 1.102–1.364; *p* < 0.001) as a continuous variable remained independently associated with the primary composite outcome.

### 3.3. Event-Free Survival

**Figure 2:**
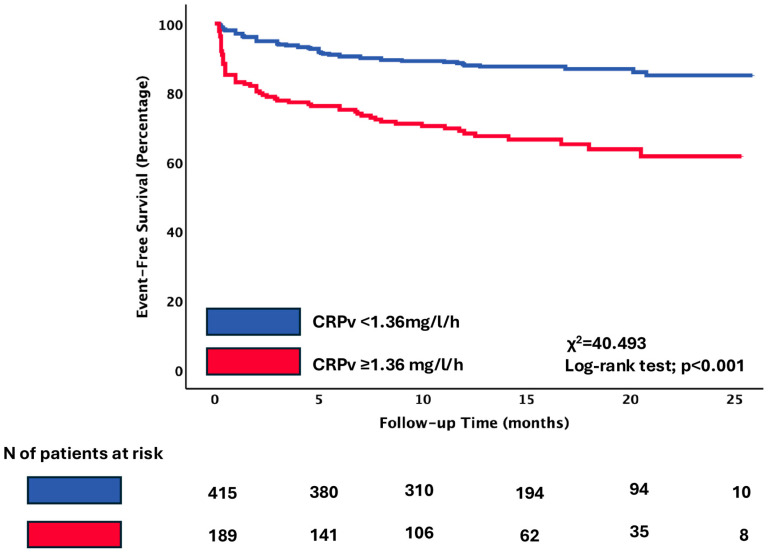
Primary composite endpoint of cardiovascular death, non-fatal AMI, and hospitalization for heart failure according to the cut-off of CRPv. Kaplan–Meier curves indicating the event-free survival rate of patients with CRPv ≥ 1.36 mg/L/h compared to patients with CRPv < 1.36 mg/L/h. Abbreviations: CRPv, C-reactive protein velocity.

Kaplan–Meier survival analysis demonstrated a significantly lower event-free survival rate for patients with CRPv ≥ 1.36 mg/L/h compared with those with CRPv below the threshold of 1.36 mg/L/h (66.7% vs. 85.5%, log-rank *p* < 0.001). The separation between the curves was evident early during follow-up and persisted throughout the study period (Figure 2).

### 3.4. Incremental Prognostic Value of CRPv for the Primary Composite Endpoint

**Figure 3:**
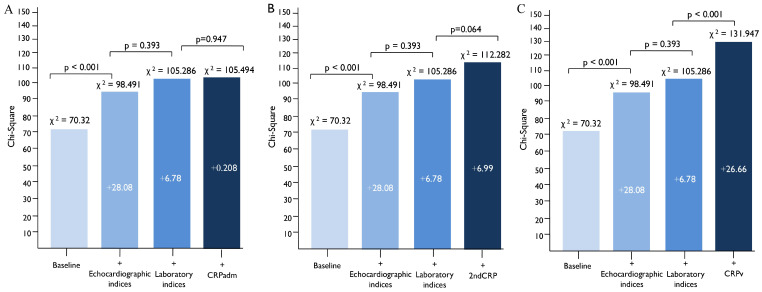
The incremental prognostic value of CRPv for the prediction of the primary composite endpoint of cardiovascular death, non-fatal AMI and hospitalization for heart failure, by comparing models including clinical, echocardiographic and laboratory parameters that affect the outcome. The addition of CRPv (**C**) provided additional prognostic value when added on top of a baseline clinical, echocardiographic and laboratory model, while the addition of CRPadm (**A**) and 2ndCRP (**B**) did not. Baseline: GRACE, history of hypertension, history of type 2 diabetes mellitus. Echocardiographic indices: LVEF, Mean E/e’, TAPSE, indexed LA volume. Laboratory indices: Creatinine, Hemoglobin, NT-proBNP, hs-cTnT. Abbreviations: CRPadm, C-reactive protein on admission; 2ndCRP, Second CRP measurement at 24 ± 8 h; CRPv, C-reactive protein velocity; MI, myocardial infarction; GRACE, Global Registry of Acute Coronary Events; TAPSE, tricuspid annular plane systolic excursion; LA, left atrium; hs-cTnT, High-sensitivity cardiac troponin T; NT-proBNP, N-terminal pro-brain natriuretic peptide.

To demonstrate the incremental prognostic value of CRPv, a baseline clinical model was created including GRACE risk score, history of hypertension and history of type 2 diabetes mellitus. The sequential addition of echocardiographic parameters (LVEF, TAPSE, Mean E/e’ and indexed LA max volume) further increased the prognostic value of the model. The further addition of laboratory parameters (NT-proBNP levels, hs-cTnT, creatinine and hemoglobin) did not significantly increase the ability of the model to predict the primary composite endpoint. However, the addition of CRPv on top of these biochemical parameters significantly improved the performance on this model (*p* < 0.001) in comparison to the CRPadm (*p* = 0.947) and the 2ndCRP (*p* = 0.064), which did not (Figure 3).

## 4. Discussion

This is the first prospective cohort study shedding light into the long-term clinical impact of the rate of rise in CRP immediately post-event in patients with first AMI. Its main findings are summarized as follows: (1) out of 604 patients, 189 (31.3%) had a high rate of rise in CRP post-AMI, as defined by the prognostic cut-off value of CRPv ≥ 1.36 mg/L/h derived from spline curve analysis, (2) higher values of hs-cTnT and NT-proBNP were independently associated with CRPv ≥ 1.36 mg/L/h, (3) patients with high CRPv demonstrated significantly worse event-free survival at a median follow-up of 13.8 (8.4–18.7) months compared to patients with low CRPv, (4) CRPv emerged as an independent predictor of the primary composite endpoint, while it provided incremental prognostic information over clinical, echocardiographic, and biochemical indices while CRPadm and 2ndCRP did not.

CRPv reflects not only the absolute magnitude of systemic inflammation but also the rate of rise, which may better capture the severity of the immune response. Interleukin-6, the key regulator of CRP synthesis is rapidly activated following coronary occlusion and a steeper increase in CRP levels may indicate an exaggerated inflammatory response [10]. Holzknecht et al. supported this pathophysiological link by showing that CRPv independently predicted early left ventricular dysfunction after ST-elevation AMI, even after adjustment for several biomarkers and was also strongly associated with microvascular obstruction on cardiac magnetic resonance [11,12]. These findings suggest that higher CRPv identifies patients with a more intense early inflammatory response after first AMI. CRPv likely reflects, rather than causes, downstream processes such as larger infarct size, microvascular obstruction, and adverse remodeling, which in turn relate to worse outcomes.

Our findings add to growing evidence that CRPv provides complementary prognostic value beyond hs-cTnT, NT-proBNP, and the GRACE risk score and expand on these. Evidence suggests that the dynamic assessment of CRP depicted by CRPv offers a more comprehensive assessment of the inflammatory response in patients with AMI compared to a single CRP measurement upon admission or upon 24 h [5]. CRPv has shown prognostic significance in predicting major adverse cardiovascular events, including short-term mortality, and adverse outcomes such as left ventricular dysfunction, atrial fibrillation and acute kidney injury [7,13,14,15]. Its strong and independent association with 30-day mortality, even after adjustment for multiple confounders, highlights its utility in early risk stratification [13]. It is a simple, inexpensive, and widely available biomarker and it requires only two measurements within 24 h, making it feasible in routine practice. Noteworthy, the early prognostic separation between low and high CRPv groups underscores its potential role in real-time bedside risk assessment. In our study we expand upon these findings by demonstrating that the prognostic value of CRPv is retained beyond the early post-AMI period and beyond a year.

While high-sensitivity CRP has been recognized as a cardiovascular risk stratification biomarker, its role in secondary prevention remains uncertain. Current studies emphasize that, despite the presence of strong evidence, there are no formal recommendations for the measurement of high-sensitivity CRP post-AMI. Its clinical utility is still limited by high intra-individual variability and lack of guideline-directed actions [16]. Moreover, in real-world practice, cardiologists and nephrologists raise concerns regarding high-sensitivity CRP testing how the results should influence further management of patients with AMI [17]. Indeed, in PROSPECT II trial, baseline hsCRP was associated with the presence of focal high-risk plaques and diffuse coronary atherosclerosis in patients with NSTEMI but failed to consistently predict long-term outcomes [18]. Our findings suggest that a dynamic measure such as CRPv provides incremental prognostic value, which may potentially overcome some of the limitations that have hampered the adoption of static high-sensitivity CRP as a post-AMI prognostic tool.

The ability of CRPv to predict new-onset atrial fibrillation and acute kidney injury reinforces the mechanistic link between inflammation and these complications [14,15,19]. The use of CRPv highlights the clinical need to move beyond static inflammatory markers toward dynamic indices that better depict the evolving nature of systemic inflammation in AMI. Its added prognostic value over conventional established biomarkers such as cardiac troponin T further supports its potential role in enhancing risk stratification. As inflammation becomes increasingly recognized as a therapeutic target in cardiovascular disease, the integration of CRPv into routine clinical evaluation may improve prognostic accuracy and show new pathways for personalized anti-inflammatory treatment strategies.

The identification of patients with increased levels of CRPv raises the hypothesis that they may potentially benefit from targeted anti-inflammatory strategies. Large, randomized trials, including CANTOS (canakinumab) and COLCOT/LoDoCo2 (colchicine), have already demonstrated the clinical relevance of reducing inflammation after AMI [20,21,22]. In addition, CRP itself has been investigated as a therapeutic target. Ries et al. reported that selective CRP apheresis safely lowered circulating CRP and was associated with fewer major adverse cardiovascular events at 12 months [23]. Whether CRPv can serve as a refined risk stratification tool to identify individuals most likely to respond to such therapies remains an important question.

Beyond its association with adverse cardiovascular outcomes, CRPv may also be related to bleeding risk prediction after AMI. It is known that systemic inflammation contributes to vascular and platelet dysfunction, potentially predisposed to hemorrhagic complications [24]. Current clinical tools for bleeding risk stratification, such as the recently updated PRECISE-HBR score, do not incorporate inflammatory biomarkers [25]. As a dynamic index reflecting the rate of CRP rise, CRPv might therefore complement those risk models by capturing the systemic inflammatory activity that underlies both ischemic and bleeding events. Although bleeding endpoints were not systematically collected in our cohort, this could warrant future research to determine whether integrating CRPv into bleeding risk assessment could enhance the predictive accuracy of existing scores.

## 5. Limitations

Despite being a prospective study, this is a single center study conducted in Greece, and the results require further confirmation and external validation in larger, multicenter cohorts to ensure generalizability across different populations and healthcare systems. The observational design represents an inherent risk of residual confounding; although, we minimized this by adjusting for multiple relevant covariates in multivariable analyses.

We assessed incremental prognostic value using nested Cox and likelihood ratio tests. Alternative measures such as NRI/IDI were not applied and could be addressed in future studies. Also, although patients with diagnosed concomitant infection were excluded from the analysis, we may have not always determined whether CRP elevation reflected a subsequent/new-onset infection or was solely related to the inflammatory response triggered by AMI. Therefore, intercurrent infections during follow-up may have influenced outcomes. Furthermore, cardiac magnetic resonance imaging (CMR) was not systematically performed, which precluded direct correlation of CRPv with imaging-based indices of infarct size. No patients in this cohort received colchicine or canakinumab; therefore, we could not assess whether CRPv identifies individuals who may benefit from targeted anti-inflammatory therapies.

## 6. Conclusions

CRPv serves as a biomarker of independent and incremental prognostic value in patients with AMI, which may lead to a timely identification of patients with augmented inflammatory post-AMI response. However, the proposed 1.36 mg/L/h threshold should be considered exploratory and requires external validation in independent, larger multicenter cohorts before clinical implementation. These patients may represent a subgroup that could potentially benefit from targeted anti-inflammatory therapies. Further prospective studies are warranted to assess both the prognostic reliability of CRPv and its potential role in selecting patients for advanced anti-inflammatory therapies and close monitoring post-AMI. Future research, including ongoing analysis within the CLEAR-AMI study, will focus on externally validating these findings across different populations and on examining whether the integration of CRPv with imaging and biochemical biomarkers can further enhance individualized risk stratification and clinical decision-making.

## Figures and Tables

**Table 1:** Baseline characteristics based on CRPv.

Variable	All (n = 604)	CRPv < 1.36 mg/L/h (n = 415)	CRPv ≥ 1.36 mg/L/h (n = 189)	*p*-Value
**Clinical Characteristics**				
Age, years	62.02 ± 11.84	60.88 ± 11.27	64.51 ± 12.69	<0.001
Male, n (%)	477 (79)	336 (81.0)	141 (74.6)	0.075
BMI, kg/m^2^	28.03 (25.85–31.14)	27.70 (25.83–30.86)	28.69 (26.43–31.72)	0.170
Systolic blood pressure, mmHg	118.19 ± 16.13	119.50 ± 15.43	115.27 ± 17.28	0.003
Diastolic blood pressure, mmHg	72.91 ± 12.03	73.60 ± 11.24	71.40 ± 13.54	0.056
Heart rate, bpm	72.97 ± 13.42	69.64 ± 11.55	80.41 ± 14.31	<0.001
GRACE risk score	119 (101–142)	114 (97–132.5)	138 (113.5–161.5)	<0.001
STEMI	416 (68.9)	256 (61.6)	160 (84.6)	<0.001
NSTEMI	188 (31.1)	159 (38.3)	29 (15.3)	<0.001
**Past medical history**				
Dyslipidemia	168 (27.8)	119 (28.7)	49 (25.9)	0.485
Family History of CAD	105 (17.4)	78 (18.8)	27 (14.3)	0.175
Chronic Kidney Disease	10 (1.7)	4 (1)	6 (3.2)	0.079
Smoking	320 (53.1)	225 (54.2)	91 (48.1)	0.240
Atrial fibrillation, n (%)	23 (3.8)	14 (3.4)	9 (4.8)	0.408
Hypertension, n (%)	217 (36)	140 (33.7)	77 (40.7)	0.096
Type 2 diabetes mellitus, n (%)	121 (20.1)	69 (16.6)	52 (27.5)	0.002
**Echocardiography indices**				
LV ejection fraction, %	47.31 ± 9.85	49.53 ± 9.03	42.39 ± 9.85	<0.001
Mean E/e’ ratio	10.41 (8.46–13.76)	9.70 (8.09–12.38)	12.35 (9.80–15.43)	<0.001
TAPSE	19.71 ± 3.27	20.24 ± 3.00	18.52 ± 3.53	<0.001
LA volume indexed, mL/m^2^	31.8 (27.22–38.45)	31.25 (27.21–37.09)	34.48 (27.56–39.39)	0.024
**Laboratory indices**				
NT-proBNP, pg/mL (range)	1087 (455–2689)	797 (349–1583)	2572 (1245–7062)	<0.001
Admission troponin T-HS, pg/mL (range)	522 (71–2554)	279 (52–1565)	1588 (254–4569)	<0.001
CPK, U/L (range)	279 (119–999.50)	211 (108.5–669)	799 (293–2187.5)	<0.001
Creatinine, mg/dL	0.97 (0.83–1.14)	0.94 (0.82–1.07)	1.04 (0.885–1.29)	<0.001
Hemoglobin, g/dL	14.00 ± 1.81	14.15 ± 1.69	13.65 ± 2.02	0.004
Platelets, K/dL (range)	253.77 ± 75.77	253.76 ± 71.44	253.80 ± 84.95	0.996

Continuous variables are reported as median (IQR), or mean ± SD and categorical variables as percentages. Abbreviations: CRPv, C-reactive protein velocity; BMI, body mass index; GRACE, Global Registry of Acute Coronary Events; STEMI, ST-elevation Myocardial Infarction; NSTEMI, non-ST elevation myocardial infarction; LV, left ventricular; TAPSE, tricuspid annular plane systolic excursion; LA, left atrium; CPK, creatine phosphokinase; hs-cTnT, High-sensitivity cardiac troponin T; NT-proBNP, N-terminal pro-brain natriuretic peptide.

**Table 2:** Univariable and multivariable linear regression analysis to identify parameters associated with CRPv ≥ 1.36 mg/L/h.

	Univariable			Multivariable		
Variable	OR	95% CI	*p*-Value	OR	95% CI	*p*-Value
Age, years *	1.026	1.011–1.042	<0.001			
GRACE risk score	1.026	1.020–1.033	<0.001	1.006	0.996–1.016	0.256
STEMI	3.427	2.202–5.333	<0.001	1.640	0.837–3.216	0.150
Smoking	0.784	0.556–1.107	0.167			
Hypertension	1.350	0.947–1.925	0.097			
Type 2 Diabetes Mellitus	1.903	1.262–2.870	0.002	1.421	0.802–2.519	0.228
Dyslipidemia	0.871	0.590–1.284	0.485			
Atrial Fibrillation	1.432	0.609–3.370	0.411			
LV ejection fraction, %	0.925	0.906–0.944	<0.001	0.983	0.955–1.012	0.253
Mean E/e’ ratio	1.125	1.081–1.171	<0.001	1.008	0.951–1.068	0.793
TAPSE, mm	0.844	0.796–0.895	<0.001	0.953	0.886–1.025	0.198
LA volume indexed, mL/m^2^	1.022	1.004–1.040	0.015	0.999	0.976–1.023	0.952
NT-proBNP, pg/mL **	5.300	3.618–7.765	<0.001	2.229	1.241–4.002	0.007
hs-cTnT, pg/mL **	4.261	2.956–6.143	<0.001	2.552	1.520–4.286	<0.001
Hemoglobin, mg/dL	0.859	0.780–0.946	0.002	0.999	0.876–1.140	0.992
Creatinine, mg/dL	2.188	1.385–3.457	<0.001	0.857	0.570–1.290	0.461

* Age was not included in the multivariable analysis due to collinearity with the GRACE risk score. ** The logarithmic transform of NT-proBNP and hs-cTnT was introduced in this analysis. Abbreviations: CI, confidence interval; OR, odds ratio; GRACE, Global Registry of Acute Coronary Events; STEMI, ST-elevation Myocardial Infarction; LV, left ventricular; TAPSE, tricuspid annular plane systolic excursion; LA, left atrial; NT-proBNP, N-terminal pro-brain natriuretic peptide; hs-cTnT, High-sensitivity cardiac troponin T.

**Table 3:** Univariable and multivariable Cox proportional regression analysis to identify parameters associated with the primary composite endpoint.

	Univariable			Multivariable		
Variable	HR	95% CI	*p*-Value	HR	95% CI	*p*-Value
Age, years *	1.044	1.028–1.061	<0.001			
GRACE risk score	1.029	1.023–1.034	<0.001	1.012	1.003–1.021	0.009
STEMI	1.197	0.799–1.793	0.384			
Smoker	0.722	0.500–1.044	0.083			
Hypertension	1.525	1.056–2.202	0.024	1.125	0.698–1.815	0.628
Diabetes Mellitus	1.956	1.319–2.902	<0.001	1.692	1.005–2.847	0.048
Dyslipidemia	1.230	0.832–1.819	0.299			
Atrial Fibrillation	1.147	0.468–2.810	0.765			
LV ejection fraction, %	0.928	0.911–0.945	<0.001	0.959	0.933–0.987	0.004
Mean E/e’ ratio	1.105	1.075–1.134	<0.001	1.000	0.958–1.043	0.985
TAPSE, mm	0.883	0.835–0.933	<0.001	0.981	0.913–1.053	0.591
LA volume indexed, mL/m^2^	1.021	1.005–1.037	0.011	0.994	0.974–1.014	0.542
log(NT-proBNP), pg/mL **	4.104	2.877–5.854	<0.001	0.999	0.583–1.712	0.997
log(hs-cTnT), pg/mL **	1.939	1.394–2.698	<0.001	1.008	0.653–1.557	0.970
Hemoglobin, mg/dL	0.776	0.709–0.850	<0.001	0.949	0.842–1.069	0.386
Creatinine, mg/dL	1.453	1.241–1.702	<0.001	1.180	0.887–1.069	0.256
CRP Velocity (mg/L/h)	1.354	1.267–1.447	<0.001	1.226	1.102–1.364	<0.001

* Age was not included in the multivariable model, because it is included in GRACE risk score. ** The logarithmic transform of NT-proBNP and hs-cTnT was introduced in this analysis. 2ndCRP was not added to the analysis due to collinearity. Abbreviations: CI, confidence interval; HR, hazard ratio; GRACE, Global Registry of Acute Coronary Events; STEMI, ST-elevation Myocardial Infarction; LV, left ventricular; TAPSE, tricuspid annular plane systolic excursion; LA, left atrial; NT-proBNP, N-terminal pro-brain natriuretic peptide; hs-cTnT, High-sensitivity cardiac troponin T; CRP, C-reactive protein; CRPv, C-reactive protein velocity.

## Data Availability

The data presented in this study are available on request from the corresponding author.

## Abbreviations

AMI	Acute myocardial infarction
CRP	C-reactive protein
CRPadm	C-reactive protein on admission
CRPv	C-reactive protein velocity
2ndCRP	Second CRP measurement at 24 ± 8 h
GRACE	Global Registry of Acute Coronary Events (risk score)
LVEF	Left ventricular ejection fraction
TAPSE	Tricuspid annular plane systolic excursion
NT-proBNP	N-terminal pro–B-type natriuretic peptide
hs-cTnT	High-sensitivity cardiac troponin T
HR	Hazard ratio
CI	Confidence interval
